# The Impact of AI Usage on University Students’ Willingness for Autonomous Learning

**DOI:** 10.3390/bs14100956

**Published:** 2024-10-16

**Authors:** Ling Wang, Wenye Li

**Affiliations:** 1 Institute of Education, Nanjing University, Nanjing 210093, China; 602023120006@smail.nju.edu.cn; 2 College of Education, Yili Normal University, Yining 835000, China

**Keywords:** artificial intelligence technology, expectation-confirmation model, willingness for autonomous learning, positive emotions, digital efficacy

## Abstract

As artificial intelligence (AI) technology becomes increasingly integrated into education, understanding the theoretical mechanisms that drive university students to adopt new learning behaviors through these tools is essential. This study extends the Expectation-Confirmation Model (ECM) by incorporating both cognitive and affective variables to examine students’ current AI usage and their future expectations. The model includes intrinsic and extrinsic motivations, focusing on three key factors: positive emotions, digital efficacy, and willingness for autonomous learning. A survey of 721 valid responses revealed that positive emotions, digital efficacy, and satisfaction significantly influence continued AI usage, with positive emotions being particularly critical. Digital efficacy and perceived usefulness also impact satisfaction, but long-term usage intentions are more effectively driven by positive emotions. Furthermore, digital efficacy strongly affects the willingness for autonomous learning. Therefore, higher education institutions should promote AI technology, enhance students’ expectation-confirmation levels, and emphasize positive emotional experiences during AI use. Adopting a “human–machine symbiosis” model can foster active learning, personalized learning pathways, and the development of students’ digital efficacy and innovation capabilities.

## 1. Introduction

Prior to the advent of artificial intelligence (AI) technology, autonomous learning had already been extensively explored. Over the past decade, AI technology has experienced unprecedented exponential growth, posing significant challenges to the practices and development of higher education [1,2]. Simultaneously, research and exploration into autonomous learning within both formal and informal learning environments have deepened. AI impacts university students’ learning through personalized learning experiences, adaptive guidance, intelligent tutoring systems, immersive learning technologies, and automated content creation [3]. Notably, generative AI technology, by simulating human-like interactive communication, allows students to access enriched and personalized learning resources, enabling them to control their learning pace and steps, thus reshaping their perception of autonomous learning [4,5]. The effective use of generative AI technology for autonomous learning is becoming increasingly critical for university students to acquire skills and adapt to the rapidly changing job market, potentially leading to significant differences in their learning outcomes and employment opportunities. Essel and Vlachopoulos demonstrated through pre- and post-tests that students interacting with chatbots performed better academically than those interacting with teachers [6]. Atalas’ research further indicated that students perceived generative AI technology not only as a source of personalized learning support, offering them round-the-clock tailored resources, but also as an aid for literature searches, abstract reading, and even hypothesis generation based on data analysis [7], thereby deepening their learning based on the latest research trends [8]. These studies collectively underscore the substantial value of AI technology in higher education, particularly in its recognized potential to support university students’ learning [9]. However, existing research falls short of fully explaining the specific factors influencing university students’ willingness to engage in autonomous learning with AI technology.

This study focuses on whether university students’ willingness for autonomous learning has shifted with the advent of AI technology as a disruptive tool. Given that generative AI can be applied in both formal and informal learning environments, this research expands beyond classroom-based autonomous learning to comprehensively examine university students’ autonomous learning in both formal and informal contexts. To investigate the impact of generative AI technology on students’ willingness for autonomous learning, it is essential first to analyze their willingness to use generative AI and the factors influencing their continued use; second, to examine whether the intention for continued use affects their willingness for autonomous learning; and finally, to clarify the specific mechanisms underlying this influence. Addressing these questions will aid educators and policymakers in understanding how best to integrate these technologies into higher education to support personalized learning for university students.

## 2. Literature Review and Research Hypotheses

### 2.1. Expectation-Confirmation Model and Its Limitations

The theoretical framework for this study is based on the Expectation-Confirmation Model (ECM), initially proposed by Bhattacherjee [10]. ECM has been extensively applied in various domains, including e-commerce, online learning, and technology usage [11,12,13]. The model comprises four core constructs: expectation confirmation (the extent to which an individual’s initial expectations are met after using an information system) [14], perceived usefulness (the degree to which an individual believes that using a system will enhance their performance) [15], satisfaction (an individual’s positive evaluation of their experience) [16], and continuance intention (the individual’s willingness to continue using the current system) [17]. Compared to the Technology-Acceptance Model (TAM), ECM focuses more on the psychological motivations following the initial use of technology, making it more reflective of the satisfaction experienced during the technology-usage process. Therefore, this study employs ECM to investigate the factors influencing university students’ use of generative AI technology for learning, providing insights that can help educators create conditions conducive to learning and AI technology usage in higher education.

**Figure 1:**
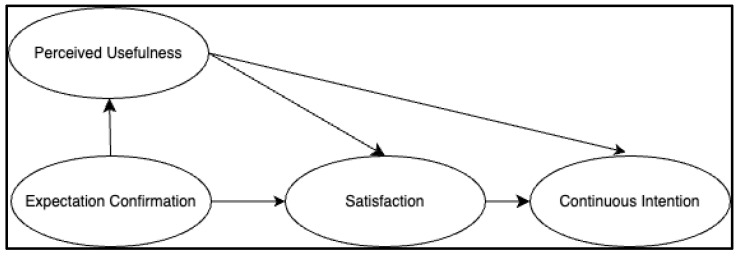
The original ECM.

As illustrated in Figure 1, the original model posits that an individual’s satisfaction and perceived usefulness of a technology determine their intention to continue using it. Satisfaction, in turn, is influenced by expectation confirmation and perceived usefulness, with expectation confirmation serving as a key determinant of perceived usefulness. Past research has widely employed ECM in studies related to information technology, providing a theoretical foundation for understanding university students’ satisfaction with AI technology. Generative AI, compared to other information technologies, is a more personalized, intelligent, and instantly responsive learning tool that subtly alters users’ emotions and behaviors, leading them to find value in the interaction with the technology, rather than just its intrinsic technical value. Therefore, this study incorporates new external variables involving interactions with generative AI technology into the original ECM model to verify whether the model still holds. This further addresses the factors influencing college students’ satisfaction with AI technology and the mechanisms affecting their continued use of generative AI for autonomous learning.

This research focuses on three critical external variables: digital efficacy, positive emotions, and the willingness for autonomous learning. First, digital efficacy reflects whether university students possess the innovative and exploratory characteristics required in a technologically advanced learning environment and whether they believe in their ability to master AI usage strategies to support personalized and deep learning [18]. Second, positive emotions are indicative of the appeal of generative AI technology to students and reveal the alignment between the resources provided by the technology and the students’ learning goals [19], influencing their future continuance intention. Third, while the traditional ECM and its extended models typically culminate in the formation of continuance intention, this study introduces the construct of willingness for autonomous learning to examine whether continued satisfaction and intention to use generative AI technology lead to an increase in students’ autonomous learning willingness.

### 2.2. Digital Efficacy

Self-efficacy, as a subjective factor mediating between motivation and behavior, refers to an individual’s belief in their ability to accomplish a specific task or set of tasks—a self-perception of capability [20]. In this study, digital efficacy refers to university students’ perceived competence and confidence in utilizing AI technology. This perception reflects whether students can effectively leverage AI tools to access valuable information and guidance, overcome potential limitations or misconceptions related to AI, and achieve their desired learning outcomes. Conversely, students who doubt their technological abilities may adopt surface learning approaches, focusing on rote memorization and meeting minimal academic requirements [21]. Bhattacherjee posited that expectation confirmation not only enhances individuals’ satisfaction with technology but also strengthens their digital efficacy in using the technology [10]. When university students find that the functionalities and outcomes of generative AI technology meet or exceed their expectations, they are more likely to use the technology confidently and effectively, experience higher satisfaction in their learning process, and regard AI tools as vital resources supporting their autonomous learning [22]. Similarly, in the context of online universities in South Korea, digital self-efficacy and perceived usefulness were significant predictors of learners’ satisfaction [23]. Additionally, students’ perceptions of technological innovation and their experience with it can influence their willingness to use the technology and the extent to which they integrate it into their learning processes [24]. Moreover, Zadorozhnyy and Lee demonstrated that individuals’ confidence in their digital technology skills significantly impacts their willingness to engage in second language communication during informal digital learning [25]. This suggests that digital efficacy can directly enhance students’ learning autonomy, thereby promoting autonomous learning [26,27].

Based on these findings, this study hypothesizes that expectation confirmation positively influences digital efficacy, and, in turn, digital efficacy positively impacts satisfaction, continuance intention, and the willingness for autonomous learning.

### 2.3. Positive Emotions

Positive emotions refer to the degree of engagement students have with their learning content and the positive experiences they derive from the learning process [28]. In this study, positive emotions are defined as the emotional experiences of university students when using generative AI technology for learning, including feelings of pleasure, satisfaction, excitement, and a sense of immersion. These emotions serve as intrinsic motivations that drive judgment and behavior, and they are based on the fulfillment of expectations. It is important to distinguish between positive emotions and satisfaction, as both relate to emotional experiences but differ conceptually. The key difference lies in the timing of measurement: satisfaction is fundamentally a retrospective evaluation following use [29], whereas positive emotions focus on the quality of emotions experienced during the process of interacting with the technology, acting as a motivational force throughout the experience. Therefore, positive emotions and satisfaction are theoretically distinct constructs.

Oliver’s Expectation-Confirmation Theory has established that when a consumer’s actual experience aligns with or exceeds expectations, positive emotional experiences tend to increase [30]. Previous research has concluded that students who have a positive attitude towards using ChatGPT for language learning are more likely to engage emotionally with the tool and perceive it as a beneficial resource for language acquisition [31]. Moreover, such positive emotions are associated with higher behavioral intentions to use ChatGPT [32] and strongly predict the actual use of ChatGPT for autonomous English learning outside the classroom [33].

Based on these findings, this study hypothesizes that expectation confirmation positively influences positive emotions, and, in turn, positive emotions positively impact continuance intention.

### 2.4. Willingness for Autonomous Learning

A high continuance intention to use generative AI technology among university students is likely to lead to a positive outcome: an increased willingness for autonomous learning. Willingness for autonomous learning refers to the learners’ tendency and inclination to actively set learning goals, select appropriate learning methods, and engage in self-monitoring during the learning process. This concept emphasizes students’ proactivity and self-regulation in learning, which are crucial for lifelong learning and personal development. In the age of AI, autonomous learning can fully harness the educational benefits of generative AI, thereby enhancing learners’ independence and self-sufficiency [34]. In this study, willingness for autonomous learning is defined as university students’ inclination to actively utilize generative AI technology for learning activities and planning.

Previous studies have shown that AI interventions can increase students’ enjoyment and willingness to communicate in foreign language learning while significantly reducing anxiety levels, indicating that AI positively impacts spoken language learning [35]. Such interventions can guide students towards autonomous learning and enhance their learning motivation [36]. Moreover, students’ academic backgrounds may also play a role in understanding their willingness for autonomous learning, as prior research suggests that learning experiences can influence students’ willingness to use technology for learning [37]. Based on these findings, this study hypothesizes that university students’ continuance intention to use generative AI positively influences their willingness for autonomous learning and that willingness for autonomous learning varies across students with different academic backgrounds.

**Figure 2:**
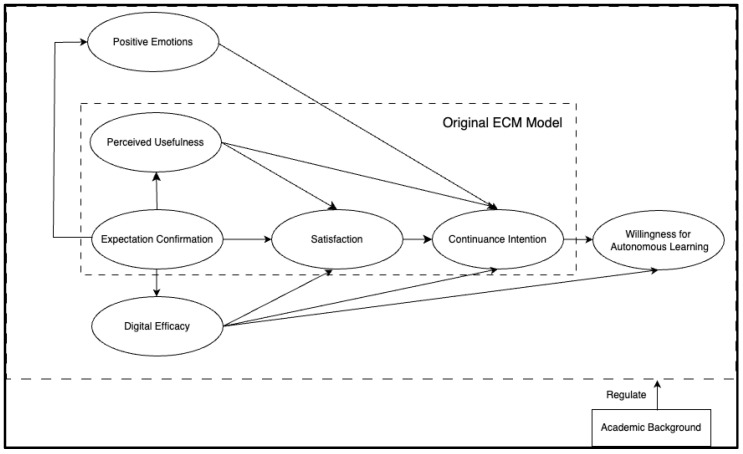
Theoretical model.

In summary, the theoretical model of this study is illustrated in Figure 2. The revised model integrates satisfaction and expectation confirmation regarding technology use, digital efficacy as a personal trait, positive emotions as intrinsic motivation, and perceived usefulness as extrinsic motivation. Through this model, the study aims to address the following four research questions:

Does the Expectation-Confirmation Model (ECM) apply to explaining university students’ satisfaction and continuance intention in using generative AI for learning activities?

Can expectation confirmation, through digital efficacy and positive emotions, enhance students’ satisfaction and continuance intention?

Does continuance intention improve students’ willingness for autonomous learning?

Does willingness for autonomous learning differ among university students from different academic backgrounds?

## 3. Research Methodology

### 3.1. Questionnaire Design

The questionnaire is divided into three sections. The first section collects basic demographic information such as gender, grade level, major, and the types of AI technologies used by the participants. The second section comprises 27 items across 8 variables, all measured using a 5-point Likert scale. These include four core variables: expectation confirmation, perceived usefulness, satisfaction, and continuance intention; one outcome variable: willingness for autonomous learning; and two newly introduced variables: self-efficacy and positive emotions. The specific items and their sources are detailed in Table 1, with item wording adapted to fit the context of this study. The third section includes an open-ended question aimed at gathering qualitative insights into university students’ experiences with generative AI technology in their learning activities, which serves to supplement and contextualize the quantitative results.

### 3.2. Reliability and Validity Testing

This study employed confirmatory factor analysis (CFA) to assess the reliability and validity of the measurement scale. Data validity was examined using MPLUS 8.3, focusing on item reliability, composite reliability (CR), and convergent validity (AVE). The results indicated that all items had reliability coefficients greater than 0.770, with squared multiple correlations ranging between 0.593 and 0.682. The lowest CR value exceeded 0.8, and the lowest AVE value was above 0.628. These findings demonstrate that the reliability and validity of the variables meet acceptable standards, thereby supporting the subsequent structural equation modeling (SEM) analysis (see Table 2).

### 3.3. Research Procedure

After completing the initial design of the questionnaire, a pilot study was conducted by inviting 35 students through a WeChat group to participate. Based on their feedback, careful revisions were made to the questionnaire to ensure that respondents could accurately complete the formal survey. The formal survey was administered online, and we established clear inclusion and exclusion criteria. The inclusion criteria required participants to be college students who had used generative artificial intelligence technology in their classroom or after-class studies and who volunteered to participate in this study. The exclusion criteria included students with a history of serious psychological illness and those unable to complete the questionnaire. To ensure the representativeness of the sample, we surveyed different types of higher education institutions and selected students from various majors and grades. Teachers distributed the questionnaire through course QQ groups or presented it via course PowerPoint presentations to invite students to participate in the survey. After obtaining consent from both the schools and the students, we explained the purpose and process of the study to them and assured them that all data would be processed anonymously to protect the privacy of the participants. During the data collection process, we obtained the necessary information through distributing questionnaires. The questionnaire included questions about demographic variables as well as other topics related to the research theme.

The online survey was conducted between 6 May 2024 and 6 June 2024. After data collection and cleaning, a total of 752 questionnaires were received. Following a screening process to eliminate invalid responses, 721 valid questionnaires were retained, resulting in an effective response rate of 95%. In terms of demographics, the sample consisted of 442 males (61.3%) and 279 females (38.7%). A total of 258 respondents (35.78%) were from Double First-Class universities, while 463 respondents (64.22%) were from regular universities. Regarding grade level, 119 were freshmen (16.5%), 131 were sophomores (18.2%), 175 were juniors (24.2%), and 296 were seniors (41.1%). The disciplines represented included 141 students (19.6%) from the humanities, 202 students (28%) from social sciences, 190 students (26.4%) from natural sciences, and 188 students (26%) from engineering. In terms of academic performance, 43.8% of the students (316) were ranked in the top 25% of their class, 28.9% (208) were ranked between 26 and50%, 17.9% (129) were ranked between 51 and 75%, and 9.4% (68) were in the bottom quartile (see Table 3).

## 4. Research Results

### 4.1. Descriptive Statistics of Variables

Descriptive statistics were calculated by summing and averaging the measurement items, yielding insights into the factors influencing university students’ intentions to use generative AI technology, as presented in Table 4. A one-sample *t*-test was conducted with a mean value of 3, representing the midpoint of the scale. A significant result indicates that the variable’s data significantly differ from the midpoint. The results show that all variables significantly differed from the midpoint, suggesting that there is notable variation in university students’ willingness to use generative AI technology for learning and autonomous study, which warrants further analysis and attention.

The mean values for each dimension (as shown in Table 4) indicate that, overall, university students had a positive experience using generative AI for learning, with perceptions across variables generally above average. Among these, satisfaction with use (M = 3.50, SD = 1.064) scored the highest, followed by positive emotions (M = 3.483, SD = 1.063), both of which were significantly above the average level. This indicates that students generally have a high acceptance of generative AI technology, maintaining positive emotions such as excitement, curiosity, and focus during its use.

Next in line were continuance intention (M = 3.472, SD = 1.089) and expectation confirmation (M = 3.470, SD = 1.052), suggesting that while students felt that the performance of generative AI in their learning largely met their expectations, it did not significantly exceed them, resulting in a moderate level of continuance intention. The mean scores for perceived usefulness (M = 3.462, SD = 1.057), willingness for autonomous learning (M = 3.276, SD = 1.069), and digital efficacy (M = 3.257, SD = 1.058) were relatively lower, indicating that some students lack sufficient confidence in using digital technology, which hinders their ability to fully leverage generative AI as a learning tool. This suggests that students’ learning habits and methods have not significantly changed, and their perceived usefulness of the technology remains modest.

The context in which students use generative AI technology may explain these findings. The survey results indicate that the primary scenarios for using generative AI are completing assignments (63.11%) and searching for necessary information (53.08%). In contrast, scenarios that involve reviewing for exams and proactively learning cutting-edge knowledge in their field accounted for only 40.36% and 41.19%, respectively. This suggests that, for many students, generative AI is a highly practical tool that aids them in efficiently completing academic tasks and gathering required materials. However, in learning contexts that require deeper understanding and autonomous exploration, students may still prefer to rely on traditional learning methods.

### 4.2. Path Analysis of the Structural Equation Model

**Figure 3:**
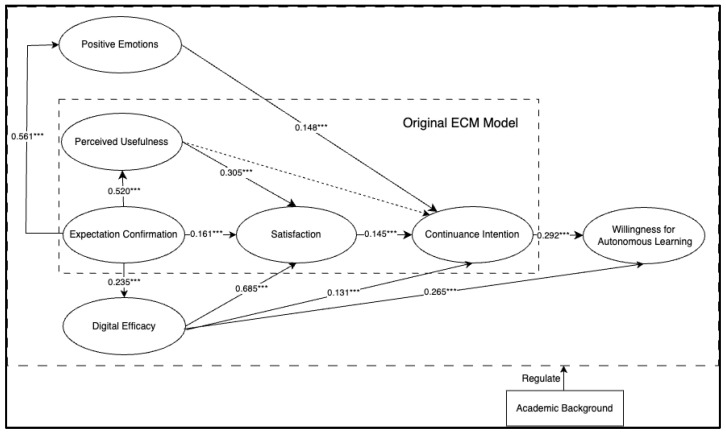
Hypothesis testing results of the final model. Note: *** *p* < 0.001.

This study employed Mplus 8.3 to construct the structural model. The model demonstrated good fit indices, as indicated by the following values: χ²/df = 3.28, CFI = 0.920, TLI = 0.900, RMSEA = 0.056, and SRMR = 0.047. These results suggest that the model’s fit indices met the acceptable criteria, indicating a good fit to the data [41]. Table 5 presents the results of the path analysis, while Figure 3 illustrates the final SEM model. The analysis revealed that four out of the five hypotheses proposed in the original Expectation-Confirmation Model (ECM) were supported, with the exception of the hypothesis that perceived that usefulness influences continuance intention. This finding suggests that the ECM is still applicable in the context of university students’ continued use of generative AI technology, addressing Research Question 1.

Additionally, all seven hypotheses related to the newly introduced variables, positive emotions and digital efficacy, were supported, thereby addressing Research Question 2. Finally, the hypothesis that continuance intention influences willingness for autonomous learning was also validated, answering Research Question 3.

### 4.3. Mediation Effects

To further address Research Question 2, this study employed the bootstrap method to examine the multiple mediation pathways from expectation confirmation to satisfaction and continuance intention. Specifically, five significant mediation pathways were analyzed. As shown in Table 6, in the pathway from expectation confirmation to satisfaction, the mediation effect of perceived usefulness was 0.161 (*p* < 0.001), with a confidence interval of (0.160, 0.256), while the mediation effect of digital efficacy was 0.051 (*p* < 0.001), with a confidence interval of (0.111, 0.206).

In the pathway from expectation confirmation to continuance intention, the mediation effect of positive emotions was 0.083 (*p* < 0.001), with a confidence interval of (0.039, 0.127), the mediation effect of digital efficacy was 0.031 (*p* < 0.001), with a confidence interval of (0.009, 0.052), and the mediation effect of satisfaction was 0.023 (*p* < 0.01), with a confidence interval of (0.005, 0.042). None of the confidence intervals for these five pathways included zero, indicating that the mediation effects are significant.

### 4.4. Moderation Effect Analysis

The analysis revealed significant differences in the impact of the continuance intention to use generative AI technology on the willingness for autonomous learning across different academic disciplines, with students from social sciences, natural sciences, and engineering showing notable variance when compared to those from the humanities. This finding addresses Research Question 5 (see Table 7). The model’s path coefficients were analyzed to further understand these differences, revealing that the moderation effect was strongest among social sciences students (0.518), followed by natural sciences students (0.442), and was weakest among humanities students (0.314).

These results suggest that students from different academic backgrounds may possess distinct cognitive frameworks. Humanities disciplines, such as literature and history, often emphasize understanding and interpreting human culture, thought, and history. The nature of these fields may lead to a more conservative approach to adopting technology for autonomous learning. In contrast, social sciences, including fields like economics, law, and management, typically stress critical thinking and the analysis of social phenomena. Research in social sciences often requires extensive data collection and analysis, areas where generative AI technology can assist students in processing large datasets and extracting valuable insights. As a result, these students recognize the potential of technology to enhance learning efficiency and quality and are more inclined to explore how it can support their autonomous learning. Students in natural and engineering sciences are accustomed to using various software tools for experiment simulation and data analysis, and while generative AI technology may not be their only tool for autonomous learning, it plays a significant role in their educational toolkit.

Similarly, the analysis of the impact of digital efficacy on the willingness for autonomous learning showed significant differences across disciplines when compared to humanities students. The moderation effect was strongest among natural sciences students (0.362), followed by social sciences (0.264) and humanities students (0.257), with the lowest moderation effect observed among engineering students (0.176). This indicates that improvements in digital efficacy significantly enhance the willingness of humanities students to use generative AI technology for autonomous learning.

## 5. Discussion and Implication

### 5.1. Discussion and Conclusions

This study extends the Expectation-Confirmation Model (ECM) to the context of university students using generative AI technology for learning, incorporating three additional variables: positive emotions, digital efficacy, and willingness for autonomous learning. By constructing a theoretical model, this research provides valuable insights into the factors influencing university students’ learning experiences with generative AI, thereby enriching the application scenarios of the ECM and offering practical implications for enhancing students’ willingness to engage in autonomous learning using AI technology.

Consistent with other research conclusions [42,43], expectation confirmation was found to significantly predict not only perceived usefulness, as proposed in the original ECM, but also the newly added variables, positive emotions and digital efficacy. The higher the degree of expectation confirmation, the higher the levels of digital efficacy, perceived usefulness, and positive emotions associated with using generative AI for learning. Expectation confirmation significantly influenced satisfaction through perceived usefulness and digital efficacy, highlighting the mediating role of these factors between expectation confirmation and satisfaction. Among them, digital efficacy emerged as the most critical factor influencing satisfaction, corroborating findings by Bai [44] and Zheng [45], who identified technological self-efficacy as a key predictor of technology use behavior and experiences.

As expected, students’ continuance intention to use generative AI technology in the future was significantly influenced by satisfaction, positive emotions, and digital efficacy. Interestingly, while perceived usefulness significantly affected satisfaction, it was insufficient to drive long-term continuance intention to use AI for learning, diverging from the original ECM. The impact of perceived usefulness on continuance intention was mediated by satisfaction, underscoring the mediating role of satisfaction between expectation confirmation and continuance intention. From a motivational theory perspective, this finding aligns with Wang and Sun’s research [46], which suggests that perceived usefulness, as an external motivator, stems primarily from students’ pursuit of efficiency in learning tools and is relatively unstable. Although it significantly impacts satisfaction, it may not consistently motivate students to continue using the technology for learning.

Among satisfaction, positive emotions, and digital efficacy, positive emotions were identified as the most critical factor influencing continuance intention. This may be because generative AI, as a virtual tutor, creates a community of inquiry through interactions with students. The more positive emotional investment students make in this community, the more they perceive the social presence of the virtual tutor, which fosters and sustains their connection, enhances learning engagement and enjoyment, and leads to meaningful learning experiences [47]. In this context, the reflective and constructive processes of knowledge acquisition through human–computer interaction bring students learning joy and reduce anxiety [48], which, in turn, stimulates their continued use of the technology.

Finally, the study confirmed that continuance intention positively influences the willingness for autonomous learning, with digital efficacy also showing a significant impact. Moreover, the research identified significant differences in the willingness to use generative AI for autonomous learning across students from different academic disciplines.

### 5.2. Implications for Practice

While AI technology has garnered significant attention from higher education institutions and society at large, with the educational sector eager to integrate it into teaching practices, it is evident that merely providing resources and technological support is insufficient. To ensure that AI technology effectively enhances student learning and development, it is essential to focus on factors such as students’ digital efficacy, positive emotions, and willingness for autonomous learning and to develop corresponding strategies and measures.

#### 5.2.1. Enhance AI Technology Promotion to Increase Expectation Confirmation

Given the importance of expectation confirmation for student satisfaction and continuance intention, universities should strengthen the promotion of AI technology to improve students’ expectation confirmation. Policymakers and university leaders need to implement clear initiatives or strategies to address the growing importance of AI in the 21st century and raise awareness among graduates of the key demands related to AI. As the “Innovation Action Plan for Artificial Intelligence in Higher Education” emphasizes, a multi-stakeholder collaborative education mechanism in the AI field should be established [49]. Based on this, universities should create public service platforms for AI science popularization aimed at youth and the general public, actively participating in outreach activities. They can also invite industry leaders as guest speakers to discuss the impact of AI on future workplaces. Additionally, universities can use various channels such as social media, campus seminars, and presentations to showcase case studies and success stories of AI applications, helping students form reasonable and positive expectations about AI technology. Teachers can enhance students’ curiosity and expectations by demonstrating the relevance of generative AI to real-world applications, such as using AI to create student profiles and academic planning, making AI technology more relevant to their real needs. Furthermore, universities should adopt diverse incentive strategies to encourage students to engage deeply with AI technology, as high levels of engagement can stimulate greater expectations and enthusiasm for future intelligent technologies.

#### 5.2.2. Foster Positive Emotions in AI Use to Achieve a “Human–Machine Symbiosis” Deep Learning Model

As AI is increasingly integrated into education, the growing autonomy of technology poses a threat to the development of human agency. To promote more autonomous and deep learning among students in the age of AI, it is crucial to enhance their intrinsic motivation rather than relying solely on extrinsic motivators like perceived usefulness [50]. There is also a need to be cautious of the phenomenon of reverse alienation caused by excessive reliance on intelligent technology. Ouyang et al. argued that the output of generative AI may not always align with individual intentions, and a forward-looking learning approach involves questioning and adjusting the content through human feedback [51]. Focusing on students’ positive emotions when using generative AI for learning can stimulate curiosity and the desire to explore, leading to higher-order thinking processes and interactions with disciplinary knowledge that are internalized as a learning interest and eventually transformed into a higher level of intellectual pursuit [52]. As Sun pointed out, individuals leverage their subjectivity to positively adapt AI technology, using its functions to meet their learning needs, thereby enabling deep learning to occur [53]. Universities should create a supportive digital learning environment where students feel confident and joyful when using generative AI, enhancing their engagement with learning under the guidance of teachers, peers, and AI agents. For instance, building AI-based learning communities can foster interaction and the sharing of experiences among teachers, peers, and students, thus strengthening students’ motivation to learn. Teachers should adhere to a student-centered educational philosophy, focusing on developing students’ higher-order thinking and enhancing their autonomy in learning. Moreover, universities should organize reflective activities on technology use, helping students to understand the potential negative impacts of over-reliance on technology, such as information silos, reverse domestication of technology, and risks of losing control over technology, thereby cultivating a proper understanding of technology and ethical behavior in its use. This approach will encourage students to maintain self-awareness, retain more autonomy, and develop a “human–machine symbiosis” learning model that leads to more efficient and deeper learning experiences.

#### 5.2.3. Cultivate Digital Efficacy to Empower More Effective Personalized Learning

Personalized learning centers on the learner, focusing on their individual needs and future development. With the advent of AI technology, all students have access to and can use it, but not all students can adapt to this technology. That is, not every student can transition from passively receiving information to actively controlling technology and resources. Personalized learning can only be achieved when learners actively control technology and resources, flexibly adjusting their learning methods to meet their independent learning needs. Therefore, in the era of intelligent technologies, enhancing digital self-efficacy provides a safeguard for the development of personalized learning among university students. As Trimmel and Bachmann mentioned, students proficient in multiple technologies tend to have higher levels of motivation in learning [54,55], and university students with higher digital self-efficacy are more likely to use technology to devise personalized learning strategies [56]. This may be because students with higher digital self-efficacy are better at recognizing and leveraging the possibilities offered by technology and can choose technological tools based on their learning contexts, values, and intentions [57]. Universities should provide ample technical resources and support to enhance students’ digital self-efficacy by focusing on the construction of smart campuses, platform operations, and creating a conducive cultural environment. They should establish clear AI usage policies and user guides to reduce students’ anxiety about using AI technologies, help students self-regulate and manage their own learning processes in informal learning spaces, and enhance their adaptability to personalized learning. In formal learning environments, teachers should pay greater attention to students’ emotional experiences, motivational beliefs, learning styles, and other non-cognitive factors. They can leverage AI technology to create online resource libraries and intelligent recommendation systems [58], recommending the most appropriate resources based on students’ learning behaviors, interests, and needs. Furthermore, students should be encouraged to participate in the creation and use of resources, fostering an active learning community that enables personalized learning.

Given the differences in how students from various academic disciplines use AI technology, universities should pay particular attention to the digital self-efficacy of humanities and social sciences students. These students need to understand the core functions and operational logic of AI technology, clarify the role of technology in their learning, and be encouraged to experiment with generative AI technologies to support their learning—without completely replacing traditional learning methods. As Miyagawa stated, in a world increasingly assisted by computers, traditional humanities skills such as idea creation and expression become even more valuable [59].

### 5.3. Limitations and Directions for Future Research

When interpreting the results of this study, several limitations should be taken into account. First, the data used in this research were collected through self-reported surveys, which may not fully capture the entire breadth and complexity of the constructs being measured. We encourage future research to collect qualitative data to cross-validate the findings. Second, our data are cross-sectional, which limits our ability to establish causal and reciprocal relationships between variables. Future studies may need to collect longitudinal data to provide a more dynamic picture of how positive emotions and digital efficacy interact to promote students’ willingness to engage in autonomous learning using AI technology. Third, the sample in this study was drawn from universities in mainland China, which may restrict the cross-cultural generalizability of the findings.

## Figures and Tables

**Table 1:** Scale items and design basis.

Latent Variable	Measurement Items	Source
Expectation Confirmation	1. The learning outcomes from using generative AI exceeded my expectations.	Adapted from Davis et al. (1989) [15]
	2. Using generative AI has enriched my learning experience.	
	3. Generative AI provides the learning resources I need.	
	4. The use of generative AI tools meets my expectations and needs.	
Perceived Usefulness	1. I believe that using generative AI enhances my learning efficiency.	
	2. I believe that using generative AI improves my learning outcomes.	
	3. I believe that using generative AI helps me achieve my learning goals.	
	4. Using generative AI aids in my deep understanding of the learning content.	
	5. Using generative AI tools is an effective use of my time.	
Satisfaction	1. I am satisfied with the benefits that generative AI brings to my learning.	
	2. The information and suggestions provided by generative AI during the learning process are useful.	
	3. Generative AI technology meets my personalized learning needs.	
	4. I am satisfied with the way generative AI interacts with me (e.g., response speed, response quality).	
Continuance Intention	1. I intend to continue using generative AI technology.	
	2. I will consider using generative AI to assist me when I have learning needs.	
	3. I would recommend generative AI technology to other students for learning.	
Willingness for Autonomous Learning	1. I am willing to use generative AI for autonomous learning.	Adapted from Davis (1989) [15] and Venkatesh et al. (2003) [38]
	2. After using generative AI tools, I am better at planning my learning activities.	
	3. After using generative AI tools, I actively seek and learn more related knowledge.	
	4. After using generative AI tools, I find it easier to find motivation and direction for learning.	
Self-Efficacy	1. I can use AI technology to obtain the information I need.	Adapted from Compeau and Higgins (1995) [39]
	2. I am confident that I can learn AI-related skills even in unfamiliar areas.	
	3. I expect that I can keep up with the advancements in AI technology and complete diverse learning tasks.	
	4. I can customize AI technology based on my learning needs.	
Positive Emotions	1. Using generative AI tools makes me more curious and interested in learning, prompting me to explore further based on specific dialogues.	Adapted from Ozkan and Koseler (2009) [40]
	2. Using generative AI tools effectively reduces my procrastination and learning anxiety.	
	3. When using generative AI for learning, I often feel positive and optimistic, even when facing challenges.	

**Table 2:** Reliability and validity of measurement variables.

Variable	Item Reliability (STD)	Internal Consistency (Cronbach’s Alpha)	Squared Multiple Correlation (SMC)	Convergent Validity (AVE)
Expectation Confirmation	0.781–0.807	0.871	0.611–0.652	0.628
Perceived Usefulness	0.779–0.813	0.898	0.606–0.661	0.639
Satisfaction	0.790–0.816	0.88	0.625–0.666	0.648
Continuance Intention	0.789–0.804	0.838	0.622–0.646	0.634
Willingness for Autonomous Learning	0.791–0.818	0.878	0.626–0.670	0.643
Digital Efficacy	0.770–0.816	0.875	0.593–0.665	0.637
Positive Emotions	0.794–0.826	0.88	0.630–0.682	0.647

**Table 3:** Demographic data of the participants (N = 721).

Demographic Variables		N	%
Gender	Female	279	38.7
	Male	442	61.3
Types of universities	Double First-Class universities	258	35.78
	Regular universities	463	64.22
Grade	Freshmen	119	16.5
	Sophomores	131	18.2
	Juniors	175	24.2
	Seniors	296	41.1
Disciplinary types	Humanities	141	19.6
	Social Sciences	202	28
	Natural Sciences	190	26.4
	Engineering	188	26
Academic Performance	Top 25%	316	43.8
	26–50%	208	28.9
	51–75%	129	17.9
	Bottom Quartile	68	9.4

**Table 4:** Descriptive statistics of variables.

Variable	Mean	Standard Deviation	Min	Max	One-Sample *t*-Test
Expectation Confirmation	3.47	1.052	1	5	88.539 ***
Perceived Usefulness	3.462	1.057	1	5	87.964 ***
Satisfaction	3.500	1.064	1	5	88.071 ***
Continuance Intention	3.472	1.089	1	5	85.64 0***
Willingness for Autonomous Learning	3.276	1.069	1	5	87.299 ***
Digital Efficacy	3.257	1.058	1	5	87.697 ***
Positive Emotions	3.483	1.063	1	5	90.372 ***

Note: *** *p* < 0.001.

**Table 5:** Path analysis results of hypotheses.

Independent Variable	Dependent Variable	Estimate	Standard Error	*p*-Value	Result
Expectation Confirmation	Perceived Usefulness	0.52	0.035	0.000	Supported
Expectation Confirmation	Digital Efficacy	0.235	0.037	0.001	Supported
Expectation Confirmation	Satisfaction	0.164	0.042	0.000	Supported
Expectation Confirmation	Positive Emotions	0.561	0.034	0.000	Supported
Digital Efficacy	Satisfaction	0.216	0.034	0.000	Supported
Perceived Usefulness	Continuance Intention	−0.035	0.039	0.362	Not Supported
Perceived Usefulness	Satisfaction	0.309	0.039	0.000	Supported
Positive Emotions	Continuance Intention	0.148	0.038	0.000	Supported
Satisfaction	Continuance Intention	0.143	0.044	0.001	Supported
Digital Efficacy	Continuance Intention	0.131	0.04	0.000	Supported
Continuance Intention	Willingness for Autonomous Learning	0.314	0.035	0.000	Supported
Digital Efficacy	Willingness for Autonomous Learning	0.234	0.035	0.000	Supported

**Table 6:** Mediation effect test results.

Dependent Variable	Pathway	Effect	BootSE	BootLLCI	BootULCI
Satisfaction	Mediation Effect	0.211	0.024	0.16	0.256
	Expectation Confirmation → Perceived Usefulness → Satisfaction	0.161	0.024	0.111	0.206
	Expectation Confirmation → Digital Efficacy → Satisfaction	0.051	0.012	0.027	0.073
Continuance Intention	Mediation Effect	0.137	0.023	0.091	0.184
	Expectation Confirmation → Positive Emotions → Continuance Intention	0.083	0.009	0.039	0.127
	Expectation Confirmation → Digital Efficacy → Continuance Intention	0.031	0.011	0.009	0.052
	Expectation Confirmation → Satisfaction → Continuance Intention	0.023	0.022	0.005	0.042

Note: BootSE, BootLLCI, and BootULCL refer to the standard error, lower limit, and upper limit of 95% confidence interval of indirect effect obtained by bootstrap method, respectively.

**Table 7:** Moderation effect test results.

Pathway	Academic Background			
	Humanities	Social Sciences	Natural Sciences	Engineering Sciences
Continuance Intention → Willingness for Autonomous Learning	0.314 ***	0.518 ***	0.442 ***	0.382 ***
Digital Efficacy → Willingness for Autonomous Learning	0.257 ***	0.264 ***	0.362 ***	0.176 ***

Note: *** indicates *p* < 0.001.

## Data Availability

The data presented in this study are available on request from the corresponding author.
